# Rat Riddance

**Published:** 1859-08

**Authors:** 


### RAT RIDDANCE.

First keep your premises clean, light, airy; next, according to our own experiments, the plan which is safest, and at the same time effective, is to get some strong-scented cheese, break it in small pieces, and mix it well with finely pulverized squills; we followed this up for several nights in succession, to the end of a happy deliverance. The most that a teaspoon or two of squills could do to a person would be to occasion a wholesome vomiting, while it either kills the rats or drives them away.

Make a paste of flour, a few sweet almonds and molasses, with a few drops of aniseed, for several nights in succession, not more than they will eat up clean, then add a tea spoonful of carbonate of barytes to about a pound of the paste. None of these ingredients are hurtful to people, in any reasonable amount.

Rats are passionately fond of fresh fish; feed them on it for several nights in succession, then strew a little arsenic on the fish. But arsenic ought not to be brought into a dwelling except by a physician, and even then no more should be left than is presently taken.

Strychnine is the speediest and most effectual. But not more than one in a million could be trusted with it we fear. To that one we say, obtain two or three grains of strychnine, empty the paper containing it on two or three table spoons of meal or sugar or cheese, or minced meat, which has been strewn on a muddy piece of board, dried, stir and mix with a stick, and throw it in the fire on the spot, put the board in the centre of the room, lock all the doors, put the keys in your pocket, the next morning the dead rats will be found on the floor, having been killed before they could reach their holes. Burn the block instantly, its having been soiled or made muddy would make it less likely to be touched by children. After mixing the poison and after burning the block, wash the hands thoroughly in warm water with soap.

